# A Review of Programs That Targeted Environmental Determinants of Aboriginal and Torres Strait Islander Health

**DOI:** 10.3390/ijerph10083518

**Published:** 2013-08-09

**Authors:** Leah Johnston, Joyce Doyle, Bec Morgan, Sharon Atkinson-Briggs, Bradley Firebrace, Mayatili Marika, Rachel Reilly, Margaret Cargo, Therese Riley, Kevin Rowley

**Affiliations:** 1 Onemda VicHealth Koori Health Unit, Centre for Health and Society, Melbourne School of Population & Global Health, The University of Melbourne, Carlton, VIC 3010, Australia; E-Mails: leah@unimelb.edu.au (L.J.); jdoy6@bigpond.com (J.D.); bradleyf@unimelb.edu.au (B.F.); mayatili@hotmail.com (M.M.); rachel.reilly@sahmri.com (R.R.); rowleyk@unimelb.edu.au (K.R.); 2 Centre of Excellence in Intervention and Prevention Science, Carlton, VIC 3053, Australia; E-Mail: becmorgan@ceips.org.au; 3 Rumbalara Football Netball Club, Shepparton, VIC 3630, Australia; E-Mail: sharon.atkinson@unimelb.edu.au; 4 South Australian Health and Medical Research Institute, Adelaide, SA 5000, Australia; 5 School of Population Health, University of South Australia, Adelaide, SA 5000, Australia; E-Mail: margaret.cargo@unisa.edu.au

**Keywords:** indigenous health, environmental determinants, evaluation

## Abstract

*Objective*: Effective interventions to improve population and individual health require environmental change as well as strategies that target individual behaviours and clinical factors. This is the basis of implementing an ecological approach to health programs and health promotion. For Aboriginal People and Torres Strait Islanders, colonisation has made the physical and social environment particularly detrimental for health. *Methods and Results*: We conducted a literature review to identify Aboriginal health interventions that targeted environmental determinants of health, identifying 21 different health programs. Program activities that targeted environmental determinants of health included: Caring for Country; changes to food supply and/or policy; infrastructure for physical activity; housing construction and maintenance; anti-smoking policies; increased workforce capacity; continuous quality improvement of clinical systems; petrol substitution; and income management. Targets were categorised according to Miller’s Living Systems Theory. Researchers using an Indigenous community based perspective more often identified interpersonal and community-level targets than were identified using a Western academic perspective. *Conclusions*: Although there are relatively few papers describing interventions that target environmental determinants of health, many of these addressed such determinants at multiple levels, consistent to some degree with an ecological approach. Interpretation of program targets sometimes differed between academic and community-based perspectives, and was limited by the type of data reported in the journal articles, highlighting the need for local Indigenous knowledge for accurate program evaluation. Implications: While an ecological approach to Indigenous health is increasingly evident in the health research literature, the design and evaluation of such programs requires a wide breadth of expertise, including local Indigenous knowledge.

## 1. Introduction

The environment in which individuals and populations live exerts a powerful effect on their health. For Aboriginal People and Torres Strait Islanders, colonisation has made the physical and social environment particularly detrimental. Effective interventions to improve population and individual health outcomes require environmental change as well as strategies that target individual behaviours and clinical factors. This is the basis of implementing a social ecological approach to health programs and health promotion [1]. Interventions following an ecological approach emphasise the relationship between people and the physical and social systems within which they live, including their social networks, organisations, communities, societies and public policies. According to ecological theory, projects that intervene at many levels offer greater potential for promoting health effectively than do those with a single focus [2,3,4]. Ecological analysis seeks to explain the complexity of health programs and the reciprocal determinism between environment and health behaviour, by drawing on core principles of Miller’s “Living Systems Theory” [3,5,6].

An ecological approach is implicit in the National Aboriginal Health Strategy definition of health [7] and environmental influences on Aboriginal health are increasingly considered in jurisdictional and research reports [8,9,10]. Furthermore, a guiding principle of the current National Strategic Framework for Aboriginal and Torres Strait Islander Health 2003–2013 is that Governments adopt a holistic approach “recognising that the improvement of Aboriginal and Torres Strait Islander health status must include attention to physical, spiritual, cultural, emotional and social well-being, community capacity and governance” [11]. It also allows Aboriginal health programs to be placed in a human rights framework in addition to a clinical one [12,13]. As Indigenous priorities and worldviews become increasingly incorporated into research and evaluation, for example through the application of the National Health and Medical Research Council (NHMRC) criteria for Indigenous health research [14], and as an ecological approach to health promotion gains acceptance [15], more and different types of health program targets, including environmental determinants, may become apparent in program design and reporting.

With this in mind, we reviewed the Aboriginal health literature to identify reports of programs that have targeted environmental determinants to date, and sought to characterise the various levels at which they operate. Given potential differences in Western and Indigenous worldviews of the purpose and aims of health programs, we present interpretations of the program targets from both perspectives.

## 2. Methods

### 2.1. Literature Search

We searched for relevant peer-reviewed journal articles (the evidence generally considered to be the most scientifically rigorous) through PubMed. Key words included (Indig OR Aborig*) AND (Intervention OR ecological OR environment) AND Australia*. Once we gathered papers that related to certain key areas, and after discussions with colleagues working in the field of Aboriginal and Torres Strait Islander health, we then searched using additional key word criteria for specific program types: tobacco; petrol sniffing; housing; school canteen; skin; financial; prison; diabetes; racism; hygiene; and natural resource management. Further searches of University of Melbourne Library, Indigenous Health InfoNet, and World Wide Web search engines including Google and Live Search did not reveal any further relevant reports. For inclusion in this review, the types of programs were restricted to those that directly targeted one or more environmental (as opposed to exclusively individual or client-level) determinants. The parameters for defining an environmental intervention were set around what was directly affected, or intended to be affected, in terms of physical, political, social and cultural factors. As Richard *et al*. state, “In addition to intrapersonal determinants of behaviors, the ecological approach… emphasises various facets of persons” environments: social networks, organizations, communities and public policies” [3]. Programs that targeted only individual-level factors, e.g., drug trials, health education *etc*. were excluded, as were those that targeted the interpersonal environment but not higher levels of the built, social or political environment (see *Identifying and Characterizing Program Targets section*). Education and training programs specifically targeting organisational capacity or processes were considered to have environmental targets for the purpose of this review, as were programs where people were moved to an alternative environment (usually Homelands). The search strategy identified 810 journal articles, of which 780 were excluded because they were not focused on Indigenous Australians, did not describe interventions, or did not include environmental determinants as a program target. In recognition of the importance of connection to Country as a determinant of Indigenous health, and in response to peer review, we have included several articles from the natural resource management literature that describe Caring for Country programs. These articles were sourced from Google Scholar in order to further illustrate those related activities described in the health research literature. They do not represent a comprehensive review of the natural resource management literature as this was outside the scope of the present work. However we noted a degree of data saturation with respect to the activities reported as part of Caring for Country.

### 2.2. Identifying and Characterising Program Targets

**Figure 1:**
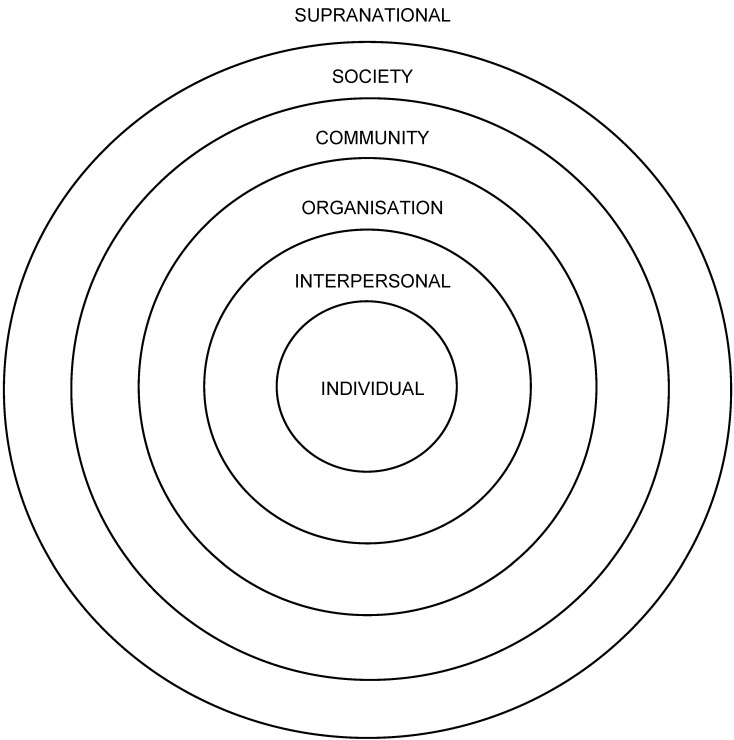
The levels of Miller’s Living Systems theory adapted for ecological analysis. Diagram adapted from Richard *et al*. [3].

Articles were reviewed for specific program activities which targeted environmental determinants of health. For characterising program targets, we referred to Miller’s Living Systems theory which defines levels at which programs can operate: individual, interpersonal, organisational, (Aboriginal and Torres Strait Islander) community, society (in the current context, the broader Australian society) and supranational (two or more countries; Figure 1) [16]. 

Coding schemes based on this theory have previously been developed as a way of characterising interventions [3,17,18]. However, using Miller’s Living Systems Theory as a framework for modelling public health activity assumes a degree of integration between the levels within a single society. This assumption is not entirely accurate in the context of a colonised and resisting Aboriginal society that in some (but not all) respects operates in parallel with mainstream Australian society rather than entirely within it. There are complex interactions between these two societies at all levels. All levels of mainstream society, from the individual level up, influence Aboriginal people’s health including through interpersonal and structural racism. Hence, from an Aboriginal standpoint, influencing mainstream society at all levels is a legitimate and necessary strategy for any efforts to improve Aboriginal wellbeing. For this reason, for simplicity, and in order to focus on the nature of Aboriginal community-based health programs, we have elected to define “society” as meaning anything outside the Aboriginal community. This means that not only political players at the national or supranational level constitute societal-level targets, but so too do any mainstream organisations (such as liquor outlets) that are the target of Aboriginal programs/activism. For this reason also we have avoided the use of the “political” category to describe targets at the societal level as used by Richard *et al*. [3,17] and by Kok *et al*. [18]. It is not clear that these coding systems were designed for the current context and this may be a limitation of the current review. However, for the purpose of indicating the breadth and complexity of the published health programs, and for providing a snapshot of the degree to which they address environmental targets, we believe it suffices. For the programs listed in Table 1, University-based researchers (L.J., B.M., K.R.) met and reached consensus, from a Western academic perspective, regarding the intended targets identified in each report. In addition, coding was performed using an Indigenous perspective by community-based researchers (J.D., B.F., S.A.-B., M.M.), most of whom also have University appointments and one of whom contributed coding specifically for the programs in and around Arnhemland, the researcher being from that region. This strategy was undertaken in recognition that Indigenous understandings of a program’s targets and purpose may differ from Western viewpoints [19], and that Indigenous researchers provide a different cultural perspective and thus reframe the research process to one that is more consistent with Indigenous worldviews [20].

## 3. Results

### 3.1. Literature Review

#### 3.1.1. Programs Identified in Peer-Reviewed Research Journals

The literature search identified an increasing number of health programs targeting environmental determinants over time. This trend was statistically significant (r = 0.542, *p* = 0.002). The earliest report identified was O’Dea’s landmark 1984 study of the effects on diabetes of returning to Traditional Country [21]. There was not another report of programs targeting people’s environment until the Minjilang program evaluation of 1994, after which the rate accelerated, with four reports appearing in 2008 and 2011. In all we found 21 different health programs (Table 1). In addition, three programs reported in the natural resource management literature are also included in Table 1. All but one program were in remote areas, mostly in the NT.

The programs identified had various aims. For the purpose of this paper these aims were categorised as: nutrition; physical activity; Caring for Country; preventing substance misuse; clinical management of chronic disease; health hardware; and social. Changes in these outcomes at an individual level were to be achieved by modifying one or more aspects of the environment in which those individuals lived. The types of environmental factors targeted included: policy; social connectedness; physical environment/infrastructure (including movement to Homelands/outstations); food supply; school curriculum; clinical practice and systems; and organisational capacity (Table 2).

**Table 1:** Program activities and targets. Targets are categorised into the following levels: IND: individual; INT: interpersonal; ORG: organisation; COM: community; SOC: Australian society (see Methods). Additional or alternative targets identified by community-based researchers are shown in bold *italics*.

Program name	Activity	Targets	Target levels
Temporary Reversion to Traditional Lifestyle (Kimberley, WA, Australia) [21] 1984 *	• Returning to traditional lands	Community members	IND, *COM*
	• Hunting/Gathering	Community members	IND, *COM*
Minjilang Health Program (Top End, NT, Australia) [22,23] 1994, 1995	• Provide and promote nutritious food in store	Food supply	IND, ORG, *INT*, *COM*
	• Regular air charter to transport fresh produce	Food supply	ORG, *COM*
	• ALPA store nutrition policy **	Food supply	ORG, *COM*
	• Elders told traditional stories highlighting overconsumption and greed and community interpreted these as warnings about overconsumption of fat and sugar.	Community members	IND, INT
	• “Shelf-talkers” to highlight target foods	Shoppers; store characteristics	IND, ORG, *INT*
	• Alcohol prohibition	Minjilang community	COM, *INT*
	• Heart health screening program	Clinical systems	ORG, *IND*, *COM*
Eliminating petrol sniffing (Arnhem Land, NT, Australia) [24] 1995	• Introduction of Avgas in Maningrida	Fuel supply	ORG, *COM*
	• Community support and governance	Community capacity	COM
	• Employment and skills-training programs	Community capacity	IND
Halls Creek Alcohol Program (Halls Creek, WA, Australia) [25] 1998	• No packaged liquor sold before midday	Hotel/Bottle shops	ORG, *SOC*
	• Cask wine only sold between 4 pm and 6 pm	Hotel/Bottle shops	ORG, *SOC*
	• One case of wine per person on any one day	Customer	IND, *COM*
	• School education program	Students	IND, *COM*
	• Introduction of CDEP	unemployed	IND, COM
	• Expanded TAFE services	Education system	COM, *SOC*
	• Arts centre established	Community infrastructure	COM
Looma Healthy Lifestyle (Kimberley, WA, Australia) [26,27] 2000, 2001	• Store management policy changes	Food supply	ORG
	• Formal and informal education sessions	Community members	IND, *INT*
	• Regular exercise groups	Community members	IND, *INT*
	• Simple dietary advice	Community members	IND
	• Sports festivals	Community members & orgs	IND, INT, ORG
	• Art competitions and sporting festivals	Community members & orgs	IND, INT, ORG
	• nonsmoking policy in public buildings	Public building space	COM, *IND*
	• Store tours to identify healthy food choices.	Shoppers	IND
	• Hunting trips, sport, walking groups	Community members, teams/groups	IND, INT, *COM*
	• Sport and recreational officer appointed	Organisational capacity	ORG
	• Council set up office as a base for program	Program infrastructure	ORG
	• Health education classes conducted by AHWs in the community school	Students	IND
	• School curriculum change	School	ORG
Nutrition awareness and healthy lifestyle program (Central Australia, NT, Australia) [28] 2000	• Changes to food supply at the community store	Food supply	ORG
	• Nutrition awareness	Community members	IND
Waste water-reuse Program (13 remote communities, WA, Australia) [29] 2001	• Evapotranspiration units installed in 13 communities	Waste management infrastructure	COM
Review of petrol sniffing programs (Aboriginal communities across Australia) [30] 2002	• Substitution of petrol with Avgas/Comgas	Fuel supply	ORG
	• Using unleaded petrol	Fuel supply	ORG
	• Locking up petrol supplies	Fuel supply	ORG
	• Adding deterrents to petrol	Fuel supply	ORG
	• Movement to outstations/homeland centres	Community members	IND, *INT*
Remote community swimming pools (Mugarinya & Jigalong, WA, Australia) [31,32] 2003, 2008	• Installation of a swimming pool	Community infrastructure	COM
	• “No School-No Pool” policy	School	ORG, *SOC*
Clinical Systems Development; ABCD (Top End, NT, Australia) [33,34,35] 2004, 2007	• Clinical guidelines	Clinical practice	ORG
	• Electronic systems to support implementation of clinical guidelines	Clinical practice	ORG
	• Staff training	Clinical capacity	ORG
Community tobacco study (Top End, NT, Australia) [36] 2006	• Smoke-free enclosed public places	Public places	ORG, COM, *IND*
	• Sports carnival sponsorship	Community members and Orgs	ORG
	• Culturally appropriate health promotion materials	Community members	IND
	• Women’s Centre tobacco education program	Women	IND
	• School education about tobacco	Students	IND
Mt Theo Program (Central Australia, NT, Australia) [37] 2006	• Placement at Outstation	Community members	IND
	• Discussion with Elders	Community members	IND, *INT*
	• Hunting	Community members	IND, *INT*
	• Love, care and pray for young people	Young people	IND
	• Education and healthcare	Community members	IND
	• Diversion program	Community members	IND
Alcohol Restrictions Trial (Alice Springs, NT, Australia) [38] 2006	• Ban on alcohol in containers >2 L	Alcohol supply	ORG, COM
	• Reduced take-away trading hours	Store	ORG, *SOC*
	• Only light beer sold in bars before noon	Hotels	ORG, *SOC*
Health Hardware Program (132 communities, TSI, NT, NSW, WA, QLD, NSW, SA, Australia) [39] 2008	• Development of survey-fix methods	Healthhabitat’s intellectual property	ORG, *SOC*
	• Local members recruited and trained	Community members & capacity	IND, COM
	• Survey fix process	Houses in community	COM
	• Fixing hardware	Family homes	INT, COM
Caring for Country (Arnhem Land, NT, Australia) [40] 2008	• Time on country	Land and people	*IND*, *COM*
	• Burning of annual grasses	Land	*IND*, *COM*
	• Using country; gathering food & medicinal resources	Land and people	*IND*, *COM*
	• Ceremony	Land and people	*IND*, *COM*
	• Protecting country/sacred areas	Land and people	*IND*, *COM*
	• Producing artwork	People	*IND*, *COM*
Homelands Movement (Central Australia, NT, Australia) [41,42] 2008, 2012	• Land Rights Act passed	Commonwealth Legislation	SOC
	• Return of Clans to traditional lands	Community members	INT, COM, *IND*
	• Establishment of outstations	Community infrastructure	COM
	• Administrative offices established	Community infrastructure	ORG, COM, *SOC*
	• Store established	Supplies	ORG, *COM*, *SOC*
	• Clinic established	Urapuntja Health Service	ORG, *IND*, *COM*
	• Outreach Health Service	Community members on Homelands	COM, *IND*, *INT*, *ORG*
	• Alcohol prohibition	Utopia community	COM
Northern Territory Emergency Response (10 remote communities, NT, Australia) [43] 2010	• 50% Income Management by Government	Aboriginal people on Social Security payments in 73 prescribed communities	IND, INT, COM
	• Racial Discrimination Act suspended	Commonwealth legislation	SOC
Health Promotion Program (Goulburn-Murray Region, VIC, Australia) [44] 2011	• Healthy canteen policy	Food supply	IND, ORG
	• Health Summer School	Health promotion practitioners	IND, ORG
	• “Hungry for Victory” youth nutrition program	U17 footballers & netballers; mentors	IND, INT, ORG
	• Provision of fruit for members	RFNC attendees, club members	IND, ORG
	• Focus groups on guidelines	Participants; organisational partnership	IND, ORG
	• Women’s Wellbeing Group	Women; organisational partnership	IND, ORG
	• 10-week body challenge	Workplace; staff members	IND, ORG
Healthy Lifestyle Program (Arnhemland, NT, Australia) [16] 2011	• School canteen with good facilities, nutritious snacks	Food supply	ORG, *IND*
	• Community marke—access to fresh fruits, bush foods, fish and shell-fish	Food supply	COM
	• Restrictions on deep-fried food sales	Store	ORG, COM
	• Healthy breakfast program at school	Students	IND, INT, ORG
	• Family food gardens	Families	INT, *IND*
	• Community footy league established	Young men	IND, *COM*
	• Healthy Lifestyle Festival	Community members	IND, INT, ORG, COM
	• Weekly walking program	Community members	IND, INT
Cape York SRS (Cape York, QLD, Australia) [45] 2011	• Govt regulated legal availability of alcohol for sale, in partnership with Elders and locals	Law	SOC
	• Individual possession limits	Community member	IND
	• Police and judicial enforcement	Community member	IND
Housing Program (10 communities, NT, Australia) [46] 2011	• Construction of new houses	Housing infrastructure	COM, *SOC*
	• Uninhabitable houses earmarked for demolition	Housing infrastructure	COM, *SOC*
Bush food harvesting (central Australia, NT, Australia) [47] 2011	• Burning	Land	*IND*, *COM*
	• Ceremony	Land and people	*IND*, *COM*
	• Protecting country/sacred areas	Land and people	*IND*, *COM*
	• Family harvesting trips	Land and people	IND, INT, *COM*
	• Processing and selling plant produce	Produce, people, family, community	IND, *COM*
Arafura Rangers (Arnhemland, NT, Australia) [48] 2012	• Burning	Land	*IND*, *COM*
	• Protecting country/sacred areas	Land and people	*IND*, *COM*
	• Training in aquaculture	Workforce capacity	IND, *COM*
	• Recording Traditional Ecological Knowledge	*unclear from description*	
Wunambal Gaambera Healthy Country Project (Kimberley, WA, Australia) [49] 2012	• Time on country	Land and people	*IND*, *COM*
	• Burning	Land	*IND*, *COM*
	• Using country	Land and people	*IND*, *COM*
	• Protecting Country/sacred areas	Land and people	*IND*, *COM*
	• Producing artwork	People	*IND*, *COM*
	• Native Title application	Legal recognition by mainstream	SOC
	• Developing partnerships with government and NGOs	WGHC Project	ORG

* Year of publication; ** ALPA: Arnhem Land Progress Association.

**Table 2:** Environmental determinants targeted in programs, stratified by program aim and levels targeted. Levels are based on Miller’s Living Systems theory (see Methods) and allocation of targets to levels are approximate only, as context varies between programs and interpretations differ.

Program aims						
*Nutrition*	*Physical activity*	*Social*	*Caring for Country*	*Preventing substance misuse*	*Clinical management*	*Health hardware*
*Society and/or community level targets*						
National policy	Local policy	National and local policy	National policy	National and local policy		
Infrastructure	Infrastructure	Infrastructure	Landscape	Infrastructure	Infrastructure	Infrastructure
Homelands living	Homelands living	Homelands living	Homelands living	Homelands living	Homelands living	
*Community and/or organisation level targets*						
Food supply		Food supply	Food supply	Supply restriction		
Transportation		Clinical systems			Clinical systems	
Organisational partnerships	Organisational partnerships	Organisational partnerships	Organisational partnerships		Organisational partnerships	
Workforce	Workforce		Workforce	Workforce	Workforce	Workforce
capacity	capacity		capacity	capacity	capacity	capacity
*Interpersonal and/or individual level targets*						
	Opportunities for exercise	Opportunities for exercis	Opportunities for exercis	Opportunities for exercis		
Social connectedness	Social connectedness	Social connectedness	Social connectedness	Social connectedness		
Diversion of spending			Generating income	Diversion of spending		
Knowledge & education	Knowledge & education	Knowledge & education	Knowledge & education	Knowledge & education	Knowledge & education	Knowledge & education

#### 3.1.2. Nutrition

Programs for improving nutrition targeted a range of environmental determinants, including food supply, workforce capacity, policy, and family (Table 2). Changes in food supply were achieved through targeting remote stores [16,22,23,26,27,28,50], canteens at schools [16,26] and a sporting club [44], the last incorporating the “Fruit Share” program that provided fruit for club members on training nights. These activities were supported by staff development through health promotion training [44], and support from local councils in the form of air charters [23] and provision of office space for project workers [26]. Breakfast programs for children were reported [26,44], as was education through school curriculum change [26], the engagement of Elders [23], and other education strategies such as “shelf talkers” and store tours to aid community members’ recognition of target foods [23,26,28]. A Healthy Lifestyles project in north-east Arnhemland also included a family food garden program that involved staff working with families to establish backyard gardens, a community market which allowed access to fresh fruits, bush foods, fish and shell-fish, and a policy of not selling deep-fried takeaway foods until at least 11 am [16]. In an evaluation of the effects of returning to traditional Country away from an urban environment, major benefits of the associated changes in diet and lifestyle on diabetes control were identified [21] and similar benefits are likely to have accrued from other programs that included Homelands living [37,41]. In an attempt to divert spending away from tobacco and alcohol to healthier dietary choices, income management for all Aboriginal people on social security payments was made mandatory under the Northern Territory Emergency Response [43], This required change at the Federal Government level by suspending the *Racial Discrimination Act 1975* to allow its implementation [51].

#### 3.1.3. Physical Activity

Like the interventions addressing nutrition, those seeking to increase physical activity targeted a range of environmental determinants including workforce capacity, social connections and community infrastructure (Table 2). Festivals incorporating sporting events were part of several health programs [16,26,27,36] and the establishment of regular sports teams or walking groups were also reported [16,44]. *Looma Healthy Lifestyle* also included regular hunting trips and the appointment of a Sport and Recreation Officer [26]. Community tobacco interventions used sports carnivals as a means of promoting anti-smoking messages [36].

Developing infrastructure for physical activity was reported as a strategy for several programs. Rumbalara Aboriginal Co-operative ran a workplace-based exercise program aimed at employees which was supported by purchase of exercise equipment, and facilitated by a partnership with another community-controlled organization [44]. By installing swimming pools in remote communities in Western Australia, the Royal Life Saving Society of Australia aimed to “ensure that the primary outcomes of improved child health and school attendance are achieved, and the opportunities to influence a broad range of social, health and economic outcomes are identified and strategies implemented to address them” [52]. A “No School No Pool” policy was introduced by two remote communities in Western Australia—children who attended school were given passes that permitted them to use the pool after school [31,32].

At Utopia in central Australia, decentralisation of the community through the return of clan groups to widely-dispersed traditional Homelands, although primarily for social reasons (see *Social Programs* section below), increased opportunities for physical activity through hunting, and limited access to store foods high in energy density [41].

#### 3.1.4. Social Programs

Many of the interventions identified in the literature used social programs as a means of achieving a variety of outcomes (Table 2). Cummeragunja Women’s Wellbeing Group was a program aimed at addressing social isolation, nutrition knowledge, and opportunities for physical activity [44]. It provided a social network for women of the community and a safe environment for sharing information and exercising as a group. At Minjilang, the community-initiated risk factor screening and education program included a social intervention where tribal Elders told traditional stories that highlighted overconsumption and greed and the community interpreted these as warnings about overconsumption of fat and sugar [23].

Healthy lifestyle festivals build and support inter-organisational and community linkages and thereby target the social environment. Festivals, a common activity among the programs reviewed here, incorporate social, physical, educational and nutrition outcomes and as such target the individual, interpersonal environment, organisational partnerships and community. Community members and visitors from surrounding communities received healthy lifestyle information during a four-day festival for the Healthy Lifestyle program in Northeast Arnhemland, and this provided the opportunity for families to celebrate relationships and culture [16]. Art competitions and sporting festivals based on the theme, “Fitness fights diabetes”, which were conducted to promote wide community participation in the Looma Healthy Lifestyle program, targeted environmental determinants of nutrition, physical activity, and social connection, physical and nutritional intervention categories.

The Royal Life Saving Society of Australia recognises desirable social impacts expected to result from the installation of swimming pools in remote communities [52]. This program also achieved a reduction in ear, skin and respiratory infections, a reduction of petty crime and a rise in school attendance [31,32].

In a study of strategies used by a remote community to address petrol sniffing, social interventions such as community support, governance and “shaming” were used [24]. Another community-driven social intervention aimed at prevention of petrol sniffing included the movement to outstations [37] and Homeland centres [30,53], The Homelands movement has been associated with various outcomes but primary motivations have been “social factors, including connectedness to culture, family and land, and opportunities for self-determination” [41], as well as the avoidance of conflict associated with centralised modes of living. At Utopia, these aims have been supported by the development of infrastructure such as administrative offices, schools, a store and a health service that runs an outreach service to each outstation [41,42].

#### 3.1.5. Caring for Country

The Caring for Country program in Arnhemland, “is a community-driven movement towards long-term social, cultural, physical and sustainable economic development” [40,54]. Six activities were defined: time on Country; burning of annual grasses; using Country; gathering food and medicinal resources; ceremony; protecting Country/sacred areas; and producing artwork. These activities aimed to improve the wellbeing of, and connections between, people and landscapes by targeting physical and social determinants. These activities were common to several programs reported in the natural resource management literature (Table 1 and Table 2), some of which were conducted as partnerships between community and mainstream organizations [47,48,49]. Following *Wanjina Wungurr Law* (encompassing time on country, passing on traditional knowledge and other activities associated with Caring for Country) was specified as the primary target of the Wunambal Gaambera Healthy Country Project [49]. This project was developed in conjunction with Traditional Owners’ successful Native Title claim and under the national Indigenous Protected Areas program [55]. It is likely that activities associated with Caring for Country were part of other homelands-based programs cited in the *Social programs*, *Physical Activity*, *Nutrition* and *Preventing Substance Misuse* sections. 

#### 3.1.6. Preventing Substance Misuse

Place-based alcohol restriction interventions have been initiated by communities with self governance. The outstations making up Utopia community are all dry [41], as are many remote communities in the NT [22,23], and this has been associated with lower rates of injury and death [56]. The Alice Springs Liquor Trial included a ban on alcohol in containers greater than 2 L, reduced take-away trading hours and allowed only light beer to be sold in bars before noon [38]. Similar strategies were used in other states [25,45].

Limiting availability and accessibility was also a common strategy in responding to petrol sniffing in remote communities (Table 2). Interventions impacted on the local environment by substituting petrol with Avgas/Comgas or unleaded petrol, locking up petrol supplies and adding deterrents to petrol. The significant outcomes of these interventions were a reduction in petrol sniffing and decreased blood lead levels. Overall related crime decreased after the initial introduction of Avgas at Maningrida, and employment increased [24,30,53]. The successful Mt Theo program was based on moving young people to a culturally-supportive outstation environment [37].

Tobacco cessation programs have focused less on restricting supply and more on education and raising awareness. These are primarily individual-level interventions. For example, school education and culturally appropriate health promotion materials were used in targeting tobacco use and creating awareness in the Northern Territory [36]. Smoking intervention activities targeting environmental factors have included non-smoking policies in public spaces [26,36]. Income management as part of the NTER had no significant beneficial effect on sales of tobacco [43].

#### 3.1.7. Clinical Management of Chronic Disease

Changes to clinical systems within health organisations were effective in improving service provision, medication rates and on the overall impact of disease management in some studies. The activities implemented included the adaptated version of the Assessment of Chronic Illness Care program, incorporating cultural competence, pathology management and pharmacology management components, to reflect specific features of interest in NT centres. This resulted in an overall improvement of disease management and delivery of processes of diabetes care [33,34,35]. The Minjilang project developed a risk factor screening program, initiated by the community after the premature death of two young men from heart disease [22,23]. Urapuntja Health Service provided outreach clinical services to Homeland communities [41].

#### 3.1.8. Health Hardware

A program was initiated to address the association of poor health and living environments [39]. A range of healthy living practices and the household infrastructure required to support them were determined and local communities approached and asked if they wished to participate. A survey-fix process with a standardised checklist was developed, and local Aboriginal people were recruited, trained and equipped to test and repair health hardware in the living environment. There were substantial improvements at 6 months follow up in the state of repair of household health hardware. An evaluation of a housing program to assess its effects on child health was conducted in 10 Northern Territory (NT) communities. The intervention included the construction of an average of 11 new houses per community. It also earmarked the demolition of uninhabitable houses, resulting in no net benefit for overcrowding [46]. Alternative waste water management systems were trialed by Engineers with limited success in remote communities in Western Australia [29].

### 3.2. Characterising Types of Environmental Targets and Their Frequency

#### 3.2.1. Levels at Which Program Targets Were Located—Western and Indigenous Perspectives

Environmental targets for each program were characterised with respect to the levels of Miller’s Living Systems Theory (Figure 1) according to the schema of Richard *et al.* [3] with modifications as described above in the Identifying and characterising program targets section. From a Western academic perspective, the most frequently observed targets were located at the level of organisations (Figure 1). Dietary quality improvement through changes in policy and practice at remote stores and other community organisations was a common strategy. Building organisational capacity through staff training and clinical systems development was noted, and restricting access to leaded petrol and alcohol through organisational policy and practice. Community-level targets were the next most frequently documented. These included developing infrastructure for remote communities, public policy implemented by community councils to address smoking and nutrition, and community-wide healthy lifestyle festivals. The interpersonal environment was targeted in about half of the identified programs through mentoring, group activities, family-based activities, and community festivals. A societal-level policy target was noted for several programs, for example where Commonwealth legislation was either enacted (in the case of the Land Rights Act 1976, allowing the establishment of Homelands) or suspended (Racial Discrimination Act 1975, to allow imposition of the Northern Territory Emergency Response). Although programs that involved moving individuals to alternative environments, such as O’Dea’s 1984 report of return to traditional Country [21] and the outstation-based Mt Theo program [37], were difficult to code as having other than individual-level targets using this schema, they clearly represent a radical change of environment for the participants and are included for this reason.

**Figure 2:**
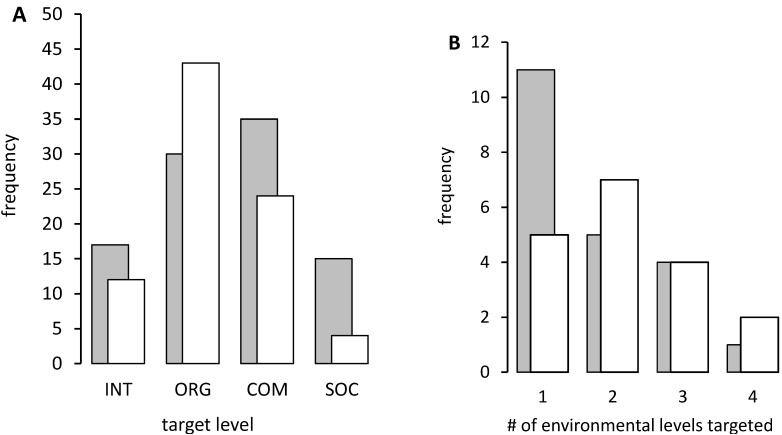
(**A**) Number of environmental targets at each level across all 21 programs identified in the health research literature, and (**B**) Number of different environmental levels targeted within programs, as interpreted by University-based (open columns) and community-based researchers (grey columns).

Community-based research collaborators, using an Indigenous perspective, more often identified interpersonal and community-level targets (Figure 2(A)). This reflected a greater focus on the role and value of relationships between people as a legitimate target for health promotion (including through such activities as group exercise or hunting), and a blurring of the boundaries between “organisation” and “community” where Aboriginal community-controlled organisations are in operation. Conversely, organisations run by mainstream Australia (such as the alcohol outlets targeted by the Alice Springs Alcohol Restrictions Trial [38], or a “no school-no pool” policy) [31] were more often placed in the category of “society”, reflecting their place outside of the Aboriginal community. Likewise, an NT community housing program was categorised as operating at the societal level due to the absence of evidence in the published report for any local community involvement in the design or implementation of the program [46]. Caring for Country, which includes the aim of “maintaining the spiritual integrity of landscapes” [40], proved particularly difficult to reconcile with Miller’s Living System Theory.

#### 3.2.2. Targetting Multiple Environmental Levels

About half of the identified programs targeted environmental determinants at two or more different levels (Figure 2(B)). The Homelands movement at Utopia community addressed all four environmental levels. This was initiated by community members who established family/clan-based outstations on traditional lands and developed community and organisational infrastructure, supported by Commonwealth legislation. It was associated with significantly lower morbidity and mortality than observed for other Aboriginal people in the NT [41].

## 4. Discussion

A relatively small number of reports appear in the peer-reviewed health research literature that describe programs targeting social and/or physical/built environmental determinants of health, although the importance of this being made explicit in current policies and frameworks is perhaps associated with an increasing number of such reports in recent years. Nutrition programs were the most often reported, and these often targeted food supply as a means of improving dietary quality for Indigenous populations. A number of interventions sought to restrict access to alcohol and petrol, and development of community infrastructure was a feature of several programs. Group exercise activities and festivals were part of several community-based health initiatives, implicitly or explicitly targeting social and cultural environmental determinants. Some of the most successful programs made use of the culturally supportive environments of traditional Country [21,37,41,54]. Many of the programs identified as targeting environmental determinants operated at multiple levels—that is, two or more of interpersonal, organisational, community and society—indicating a degree of congruence with an ecological approach to health promotion. While we did not seek to examine individual-level outcomes as part of this review, we do note that the most complex programs with multiple environmental targets achieved measurable improvements in health [22,26,27,41]. We also note that the vast majority of articles reported programs from remote areas despite most Aboriginal and Torres Strait Islander people living in urban and rural areas. Other authors have attributed this bias to unsupported assumptions about “assimilation” and the imposition of false concepts of “authenticity” onto Aboriginal people by Western researchers [57,58]. Given common determinants of health for diverse Indigenous populations, including those in urban and rural areas [59,60], all the targets identified in this review are potentially relevant beyond the remote setting.

The relative lack of programs with environmental targets appearing in the peer-reviewed health literature does not necessarily reflect a lack of effort for environmental change, and a review of the grey literature would likely reveal many more such programs. This was beyond the scope and resources of the current work which sought to characterise the nature of health research and evaluation. Within the health research literature, the expertise and priorities of researchers and program evaluators has a strong influence on the nature of information reported, as does the precision of available measurement techniques. For example, it is relatively straightforward to document rates of specific infectious conditions, the medical and epidemiological expertise to do so is readily available in the research community, and the importance of improved clinical outcomes is not disputed. Hence the evaluation of the effects of installing swimming pools in remote communities has focused on this outcome despite the other stated aims of the program that are overtly social in nature [31,32]. It is also sometimes the case that the outcomes of a program desired by community members differ from those considered a priority by program funders and evaluators. Alcohol management programs are a case in point, where a survey of over 80 Aboriginal Health Workers identified the quality of relationships between program participants and their family and community as being the overwhelming priority, well ahead of the quantity of alcohol which may be consumed [19]. Vickery and colleagues [61], using oral histories and other sources, identified a number of social determinants of Indigenous health and placed them in the context of colonisation and decolonisation, the latter being the appropriate response for reversing the damaging effects of colonisation on contemporary Indigenous health status. In addition to the social determinants of health identified by the World Health Organisation, these Indigenous authors listed history, racism, place and land, incarceration, family separation and housing as important influences on Indigenous health. Few of these social determinants of health were explicitly described in the majority of published articles reviewed here. Management of land and natural resources is a field of relevance to human ecology and health in which Indigenous researchers and knowledge are integral [62]. Burning, art, ceremony, collecting food and medicine, and protecting Country are the embodiment of culture and connection to land, spirit and ancestors for Aboriginal people. As such they cannot be separated from wellbeing. This is partly reflected in studies of the relationship of clinical and psychosocial wellbeing to involvement in Caring for Country and the appearance of Homelands living as a strategy in several successful health programs. However, most descriptions of Caring for Country programs appear in the natural resource management rather than the health literature which was the focus of this review. The health, environmental and economic effects of involvement in natural resource management have been reviewed elsewhere [63,64].

Thus, “recognising that the improvement of Aboriginal and Torres Strait Islander health status must include attention to physical, spiritual, cultural, emotional and social well-being, community capacity and governance” [11], the peer-reviewed health research literature suggests a lack of research partnerships with the combined expertise to provide comprehensive Indigenous health program evaluation. The absence of grey literature from this review does not weaken this conclusion.

Our analysis of environmental target types is necessarily imprecise in most cases, and provides only a rough guide to the complexity of reported programs. This imprecision arises in part from the differing worldviews and interpretations of Western and Indigenous perspectives, but also from a lack of local knowledge of program aims on our part. Aboriginal researchers more often considered the close relationships between individuals, families, the organisations they deal with and the communities in which they are located when identifying the intended targets of a health program activity. For example, walking groups were seen as an inherently interpersonal and public activity, with one author noting that “there is no such thing as just going for a walk”—where and with whom it takes place are important socially and culturally. Co-authors also acknowledged the limitations of relying on the content of written reports rather than local knowledge of how and why programs operate, notwithstanding that some of the published articles included local Indigenous co-authors, providing valuable local knowledge and interpretation of results [65]. Thus standardising the evaluation of Indigenous health programs may be difficult given differences in interpretation of their nature and purpose. Other authors have also highlighted the importance of including local knowledge in program design and evaluation—we agree with authors of a recent review of the ecological nature of health promotion programs for nutrition and physical activity, who stated that “the adoption of multiple perspectives and a collaborative style should be included among the core orientation underlying this research agenda” [66]. Vickery *et al*. noted the importance of Indigenous people being “part of the research and analysis to prevent determinants being reviewed through another culture’s worldview”. The research process itself is a potentially colonising act and the danger of misinterpreting the aims and desired (and observed) effects of a health program remain. Not that every aspect of a program is everybody’s business—Vickery and colleagues also note the importance of Indigenous modes of knowledge dissemination, and the issues of what, when, how, by whom and to whom information can be imparted.

The current literature review also identified several papers making recommendations for programs targeting environmental determinants of health. A need to focus on the supply and demand side of healthy food and nutrition was identified by several authors who have suggested economic interventions by including greater taxation on energy-dense, nutrient-poor food, subsidisation for healthy food, improved freight and improved remote housing infrastructure for storage and preparation of food [67,68]. An ecological approach to health promotion associated with poor hygiene and burden of infection amongst children recommended: community housing construction, repair and maintenance programs; environmental health programs including garbage collection and disposal; animal control; and maintenance of public places/sewage treatment [69].

## 5. Conclusions

Although the health research literature is somewhat limited regarding programs that targeted environmental influences on Indigenous health, examples emerged of complex, multi-level programs targeting several determinants of health. In interpreting the nature of the programs reviewed here, we were limited to the information included in published articles, which in turn is limited by the knowledge of the authors about how and why programs were implemented at a local level, the editorial policies of the publishing journals, and the parameters of what is considered valid “evidence” by the mainstream research community. Some articles included community-based and Indigenous researchers as authors, increasing the likelihood of an accurate description of the aims and targets of local interventions. The authors of this review included Aboriginal people and others of European background, and interpretation of the published information by these groups diverged in some instances—this is not unexpected given diverse worldviews and understandings of the purpose of health programs. We suggest that the design and evaluation of health programs requires a wider breadth of expertise, including local Indigenous knowledge, than is generally evident in the health research literature to date.
